# Orthotic treatment of idiopathic toe walking with a lower leg orthosis with circular subtalar blocking

**DOI:** 10.1186/s12891-021-04327-0

**Published:** 2021-06-07

**Authors:** N. Berger, M. Bauer, A. Hapfelmeier, M. Salzmann, P. M. Prodinger

**Affiliations:** 1 Children’s Orthopaedics, University Hospital Rechts der Isar, Munich, Germany; 2 Cand. med, Technical University Munich, Munich, Germany; 3 Institute of General Practice and Health Services Research, Technical University of Munich. Institute of Medical Informatics, Statistics and Epidemiology, Technical University of Munich, Munich, Germany; 4 Orthopaedics, Krankenhaus Agatharied, Agatharied, Germany

## Abstract

**Background:**

There is no universally accepted treatment standard for idiopathic toe walking patients (ITW) in the current literature. None of the established methods provide homogenous satisfying results. In our department we treat ITW patients with lower leg orthoses with a circular foot unit for a total of 16 weeks. In this study we reviewed our database to evaluate the success of our treatment protocol for a 24 months follow up period.

**Results:**

Twenty-two patients were included in this study. Age at the beginning of treatment was 7.0 years +/− 2.9 (range 2.5-13.1). Percentage of ITW at the beginning of treatment according to the perception of the parents was 89% +/− 22.2 (range 50-100). Immediately after the treatment with our device, percentage of ITW dropped to 11% +/− 13.2 (range 0-50). After 12 months, 73% of the patients (16/22) walked completely normal or showed ITW less than 10% of the day. After 24 months, 64% of the patients kept a normal gait (14/22).

**Conclusion:**

This study provides evidence that the treatment of idiopathic toe walking with lower leg orthoses with a circular foot unit results in satisfying long-term results in two thirds of the patients.

**Supplementary Information:**

The online version contains supplementary material available at 10.1186/s12891-021-04327-0.

## Introduction

Idiopathic toe walkers (ITW) are patients (typically children), who habitually walk on their toes at an age when they usually should show a physiological gait pattern. Neurologic, neuromuscular or biomechanical reasons have to be excluded. Prevalence among five to 6 year olds is about 5 % [1]. Males are slightly predominant [1].

There is evidence that habitual toe walking often resolves spontaneously in a majority of patients. Engström et al. showed that at 10 years of age 55 (87%) out of 63 children who had been former toe walkers had ceased toe walking [1].

If toe walking persists for an extended period of time, structural changes might develop [2–5]. Furthermore, the walking pattern might become socially stigmatizing for older children.

Inconsistent measuring methods exist in the literature to quantify lower limb changes for idiopathic toe walking children. Ankle joint range of motion and gait analysis are the most frequently used outcome measurements [5]. Some authors use the parents’ perception as a primary observational tool of the toe walking gait [6–9]. Gait laboratory investigations are significant but can be flawed because children sometimes tend to disguise tip toe walking patterns and show their “best gait” for the observational period [8, 10, 11].

Today, a variety of treatment options of ITW are used, including stretching exercises, serial casting, orthotic treatment, and operative procedures – all of them optional in combination with botulinum toxin injections into the calf muscles [12]. Furthermore, motor control interventions and auditory feedback methods are optional [12]. Results of these methods vary broadly and are often dissatisfying, leaving a significant proportion of “non-responders” up to 88% after 12 months [2, 7–9, 13].

In our clinic we have been treating ITW patients with a lower leg orthosis with circular subtalar blocking for several years. This construction is internationally unique because the supplying company held patents on crucial elements of the orthosis until recently. The most important feature of the orthosis is a circular foot unit, that is attached to the lower leg unit with hinges. The orthosis fulfils all functions of a cast [14]. Compulsory health insurance in Germany has acknowledged the orthosis and substitutes its costs.

The aim of this study is to provide a retrospective evaluation of the treatment of ITW with the circular lower leg orthosis at our clinic. The primary outcome parameter was the extent of gait normalisation. Secondary outcome parameters were ankle joint dorsiflexion capacity, frequency of recurrence and frequency of adverse side effects.

## Methods

We searched our in-house-database for the ICD codes “gait impairment (R26.8)”, “equinus (M21.62; Q66.8)” and “contracture of muscle/joint in lower leg/foot (M62.46; M62.47)” from 2014 to 2018 and identified patients with the diagnosis “idiopathic toe walking”. Underlying neurologic, neuromuscular or teratologic disorders were ruled out by a senior neuroorthopedics consultant (MS) and these patients were excluded accordingly. Patients with a diagnosed autism spectrum disorder were also excluded. Patients had to be at least 2 years old at the beginning of treatment. The duration of toe walking had to exist at least 6 months prior to the diagnosis. The Minimum follow-up was set to be 2 years.

### Outcome measurement

Prior to the start of the treatment and at every scheduled visit ankle joint mobility was measured in a corrected subtalar position with a neutral calcaneus position in a straight and in a bend leg, using a goniometer. Differences in passive ankle joint mobility between the right and left leg were rare. If they did exist, they were only of a small amount. We averaged their values.

At every visit, the proportional amount of time spent toe walking was assessed by asking the caregivers using a standardized question mode. The caregivers were asked to approximate the daily percentage (0-25-50-75-100) of barefoot toe walking without a sound first heel contact during the past week. Five groups corresponding to 0-25-50-75-100% were defined and patients allocated accordingly (Fig. 4). If toe walking reoccurred and orthotic treatment resumed (either with a new or the existing orthosis) patients were transferred into group 6 (recurrence group).

### Orthosis

**Fig. 1:**
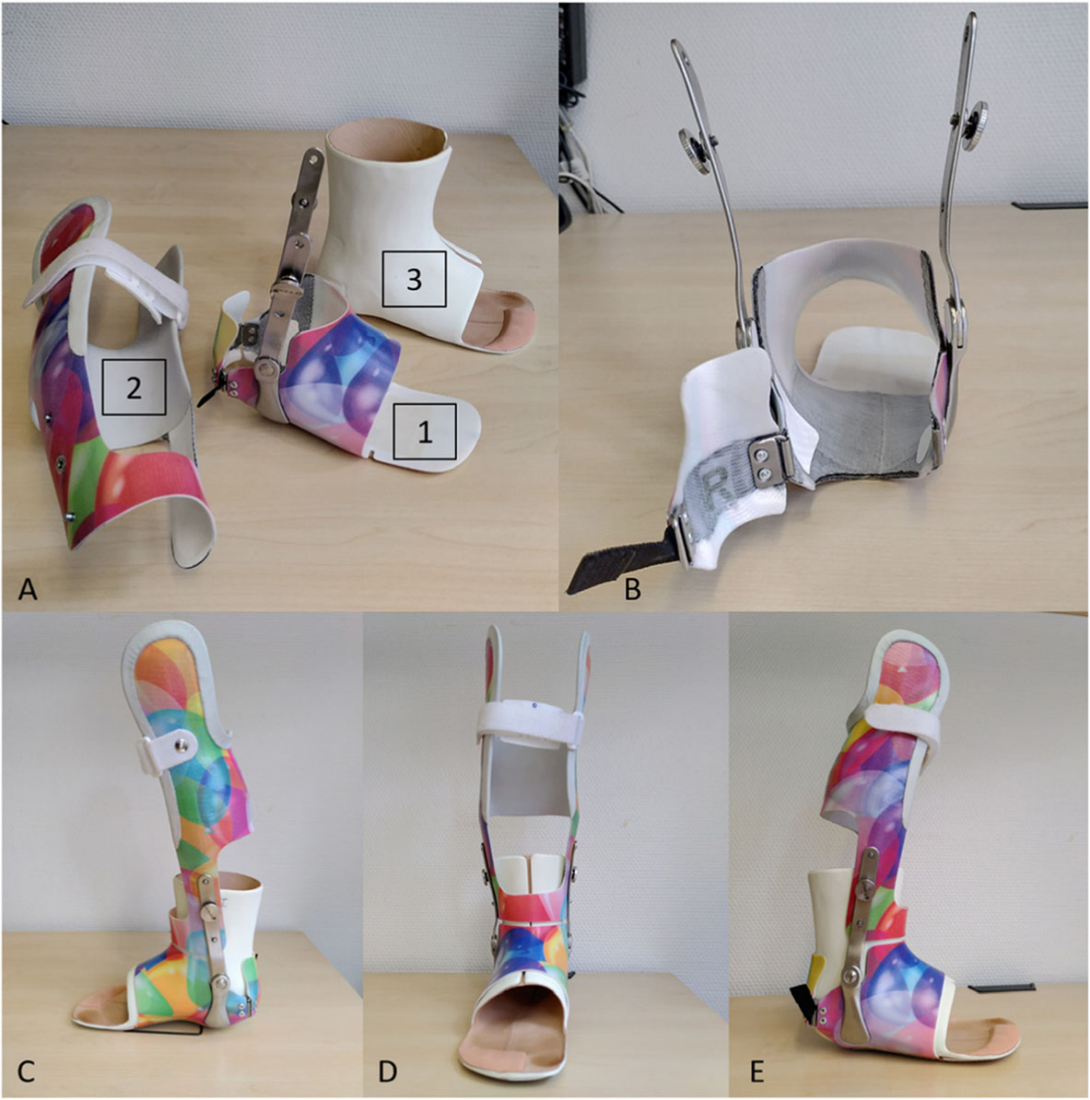
**a** The component parts are the circular foot unit (1), closed by a heel cap as seen in (**b**), the lower leg unit (2) and the liner (3). The foot is held in a slightly external, physiological rotational position (**d**). Plantarflexion and dorsiflexion can be blocked or allowed by lateral hinges, as desired (**c**, **e**)

All patients were treated with a lower leg orthosis with a circular foot unit. This unit is ring-shaped, slides over the foot from the front and is closed over the heel by a cap (Fig. 1). A liner worn under the orthosis reduces pressure points and provides padding of contact areas. The foot is encompassed in the circular foot unit with a maximum area of contact, comparable to a mounted cast. The subtalar joint is blocked. Correctional pressure forces are applied over a large area. Therefore, a displacement of the foot in the orthosis becomes less likely.

**Fig. 2:**
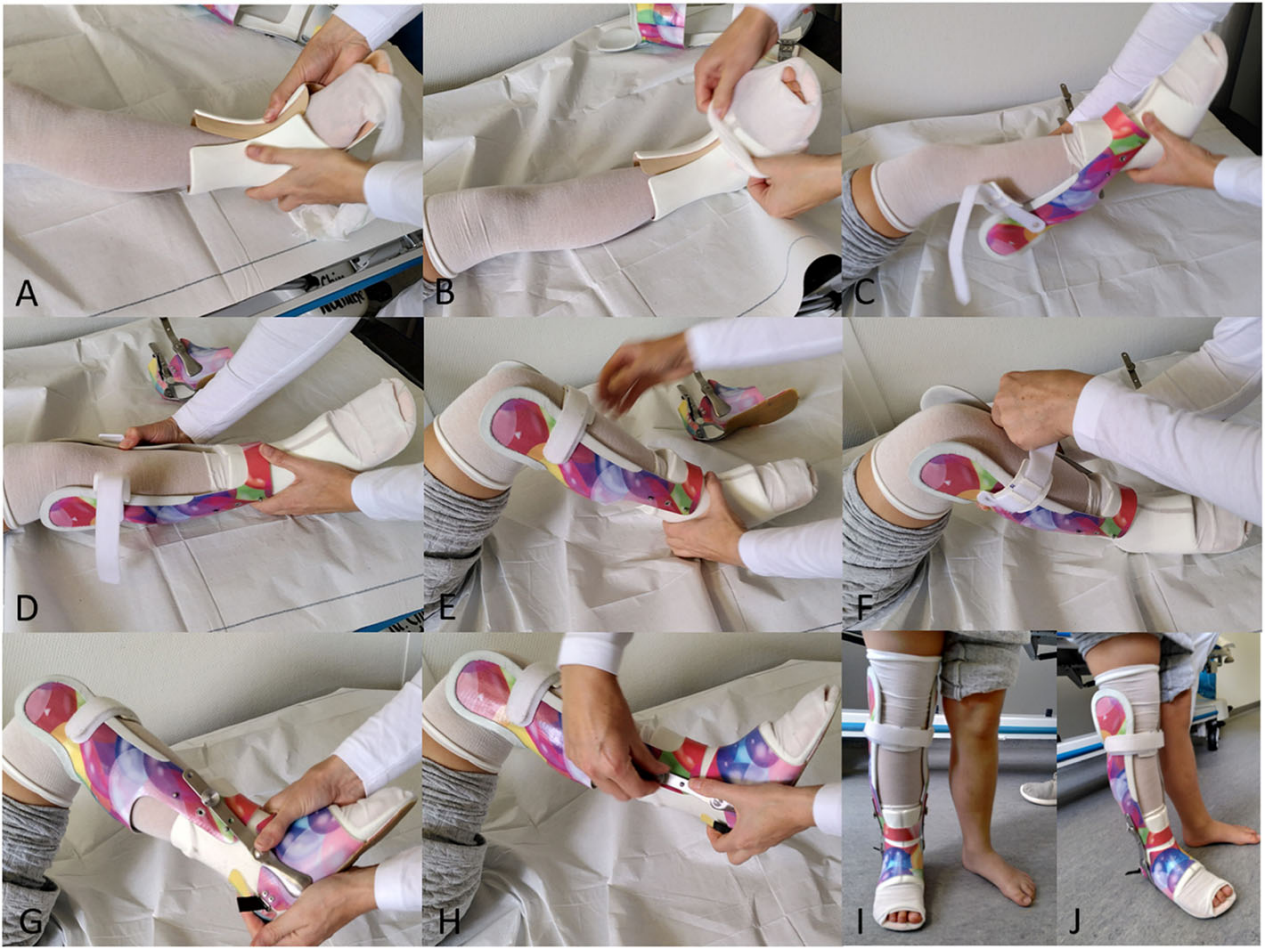
**a**-**j** Putting-on of the Pohlig lower leg orthosis: **a** stocking is put on the leg before the liner is applied, then the stocking is pulled over the liner (A + B). Now the lower leg unit is slipped over the foot and fixed (**c**-**f**). The foot unit is slipped over the foot and the heel cap is closed (**g**). The foot unit is fixed to the lower leg unit by screws (**h**). The mounted orthosis fixes the foot in a neutral dorsiflexion position and physiologic amount of external rotation. 5–10° of dorsiflexion are allowed by hinges when walking in the orthosis (I + J)

The lower leg unit is stabilized by a three-point fixation. Two lay-on points are fixed, the third one is a closing cap at the tibial tuberosity to complete the fixation. Similar to the idea of Sarmiento in 1972, the lower leg experiences an additional stabilization of the lower leg unit with an activation of muscles [15]. If desired, ear-shaped mouldings at the condyle level of the femur provide additional rotational stability (Figs. 1 and 2).

The orthosis is custom-made from a 3D-scan or a plaster cast. Its body consists of fibre-reinforced material and is therefore very rigid. The sole is more flexible due to incorporated aramid fibres. The liner is made of Tepefoam.

**Fig. 3:**
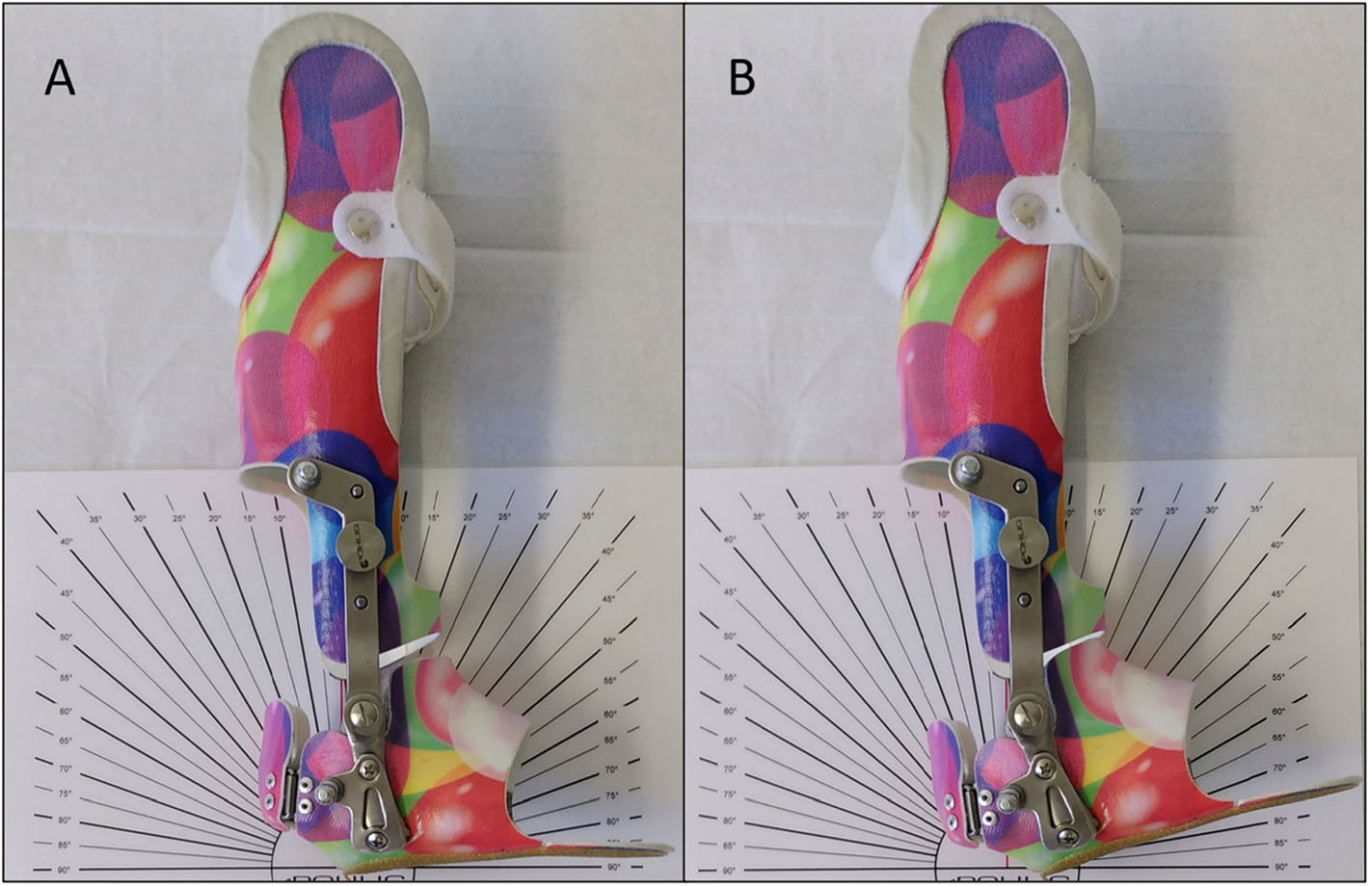
ROM of the foot unit. The lower leg orthosis in neutral position (plantarflexion is blocked); (**a**) and maximum dorsiflexion of slightly above 10° (**b**)

Hinges at the ankle joint allow for approximately 15° of dorsiflexion, plantarflexion is blocked in a neutral position (Fig. 3).

### Treatment

An optimal fit of the orthosis was guaranteed by orthopaedic technicians in an initial fitting session. Putting on and taking off the orthosis was explained to and practiced with the caregiver(s). If any problems occurred at home, a prompt follow-up visit was arranged.

During the first 6 weeks of treatment patients were instructed to wear the orthosis 23 h a day. It was only taken off for hygienic procedures and during sports/physiotherapy. The next 4 weeks, the orthosis was trained off during the daytime. For the last 6 weeks the orthosis was only worn at night-time. This point of time was defined as “end of treatment”. Beyond that period of 16 weeks, patients were free to adopt the wearing time of the orthosis to individual needs.

Physiotherapy treatment was continued if participants had already received it prior to orthotic treatment.

Visits were scheduled one and a half, 3, 6, 12, 18, and 24 months after handing out the orthoses. During every visit, the appropriate fit of the orthosis was checked. Problems with putting on and taking off were addressed. Signs of usage were checked and, if absent, noted. Patients were examined for pressure sores or skin breakdown. In addition, they were asked if it was painful to wear the brace or if they perceived pain while walking without the orthosis. Ankle joint mobility was examined as specified before.

### Statistical analysis

The distribution of quantitative data was described by mean, standard deviation and range. Hypothesis testing of group differences was performed by Mann-Whitney-U Tests. Qualitative data was presented by absolute and relative frequencies and compared between groups using Fisher’s exact test. The correlation of quantitative data was explored by Spearman’s rank correlation coefficient and the respective z-Test. A linear regression model was used to simultaneously assess the conditional effects of age and sex with respect to the improvement of toe walking. The latter was defined as the difference between the follow-up measurements at 12 or 24 months (as indicated) and the baseline measurement of the parent reported toe walking frequency of the patient. Hypothesis testing was performed at exploratory two-sided 5% significance levels. Analysation was conducted using R 3.6.1 (The R Foundation for Statistical Computing, Vienna, Austria).

### Ethics approval, consent to participate

Ethical approval was obtained by the Ethics Committee of the technical University Munich (ref. number 435/18 s). Informed consent was obtained from a parent and/or legal guardian from all subjects, as subjects were under 18. All methods were carried out in accordance with relevant guidelines and regulations.

### Results

From 2014 to 2018 we identified 224 patients with an ankle equinus. 192 were excluded from this study due to a causative congenital or a neurological disorder. Two of the remaining 32 patients were under the age of 2 years at the start of the treatment and were excluded consequently. Five patients were excluded because the follow-up time was less than 24 months, three children moved and consequently did not participate in the follow-up. The remaining 22 patients (8 f, 14 m) were included in the study.

Mean age at the beginning of treatment was 7.0 +/− 2.9 years (range 2.5-13.1). Mean percentage of toe walking during the day was 83% +/− 22.2 (range 50-100) according to the parents’ perception (Table 1).

**Table 1:** Parents were asked to estimate the time their child spent tip toeing when walking. The grouping was performed accordingly. When children were reequipped with orthoses after a treatment-free interval it was graded as recurrence (group 6)

	Percentage of toe walking
Group 1	0%
Group 2	25%
Group 3	50%
Group 4	75%
Group 5	100%
Group 6	Recurrence

### Allocation into groups according to percentage of toe walking

Before the beginning of the treatment, one patient was classified into group 3 (50%_ITW), five patients into group 4 (75%_ITW) and sixteen patients into group 5 (100%_ITW). No patients were allocated into group 1 and 2 at the beginning of treatment (for patients’ details see the supplementary file).

Once parents adapted to the handling of the orthosis – usually within 1 week –, no further problems with putting on and taking off of the orthoses occurred. Some patients reported problems with non-desired opening of the heel-cap. This problem was resolved with a technical modification. All orthoses showed signs of usage.

Mean wearing time of the orthosis was 23 weeks +/− 14.5 (range 8-73).

**Fig. 4:**
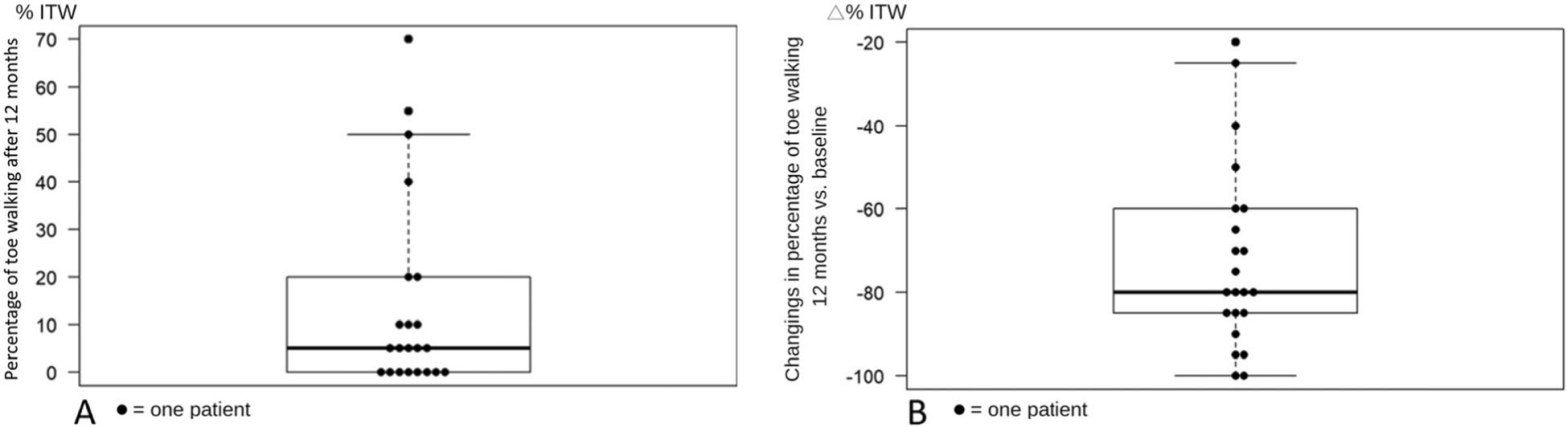
Boxplots showing the percentage of toe walking at 12 months of follow-up and changing in percentage of ITW at 12 months compared to baseline

At the end of the treatment, mean toe walking time dropped to 11% +/− 13.2 (range 0-50). One patient developed recurrent ITW (75%) after 8 months and restarted wearing the orthosis. This patient was classified into group 6 (recurrence group). All patients (including the recurrence case) improved their gait pattern after 12 months (Fig. 4).

At that point (12 months), 14 patients (64%) were classified into group 1 (0%_ITW), two patients (9%) into group 2 (25%_ITW), three (14%) into group 3 (50%_ITW) and one (5%) into group 6 (recurrence_ITW).

Deliberate night-time bracing was continued in our study group by 9 patients (41%) for an average of 18.9 weeks +/− 15.2 (range 4.6-56.7).

**Fig. 5:**
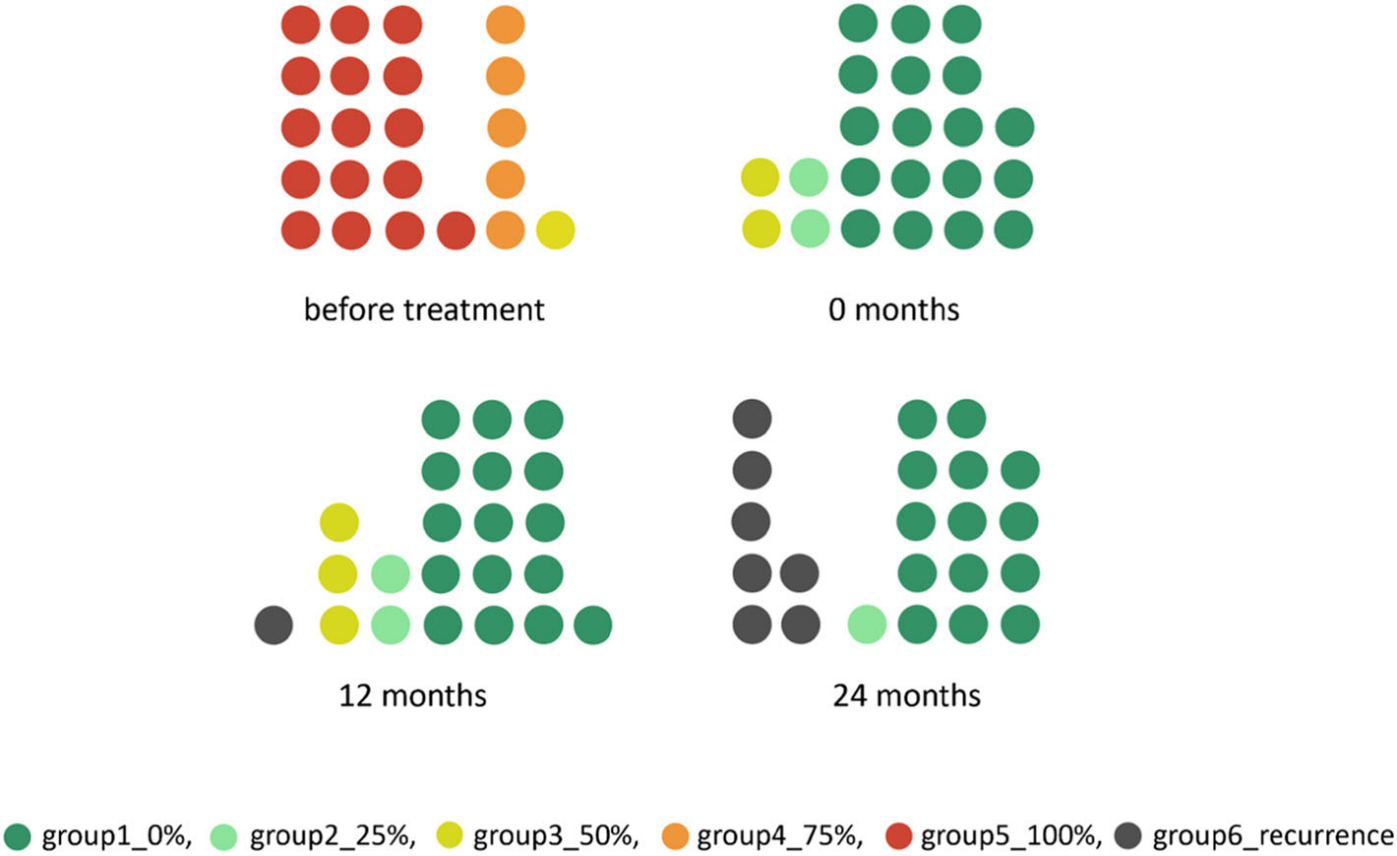
Twenty-two patients (o = 1 patient) have been grouped according to percentage of toe walking gait. Patients reprovided with orthoses were allocated into the recurrence group

During the second year of follow-up three patients suffered a recurrence and were equipped with an orthosis again. Two years after the end of the treatment 14 patients (64%) remained in group 1 (0%_ITW), one patient in group 2 (25%_ITW) and seven patients (32%) in the recurrence group (Fig. 5). At this point, the average age of our patients was 9.1 years +/− 2.9 (range 4.5-13.1).

**Fig. 6:**
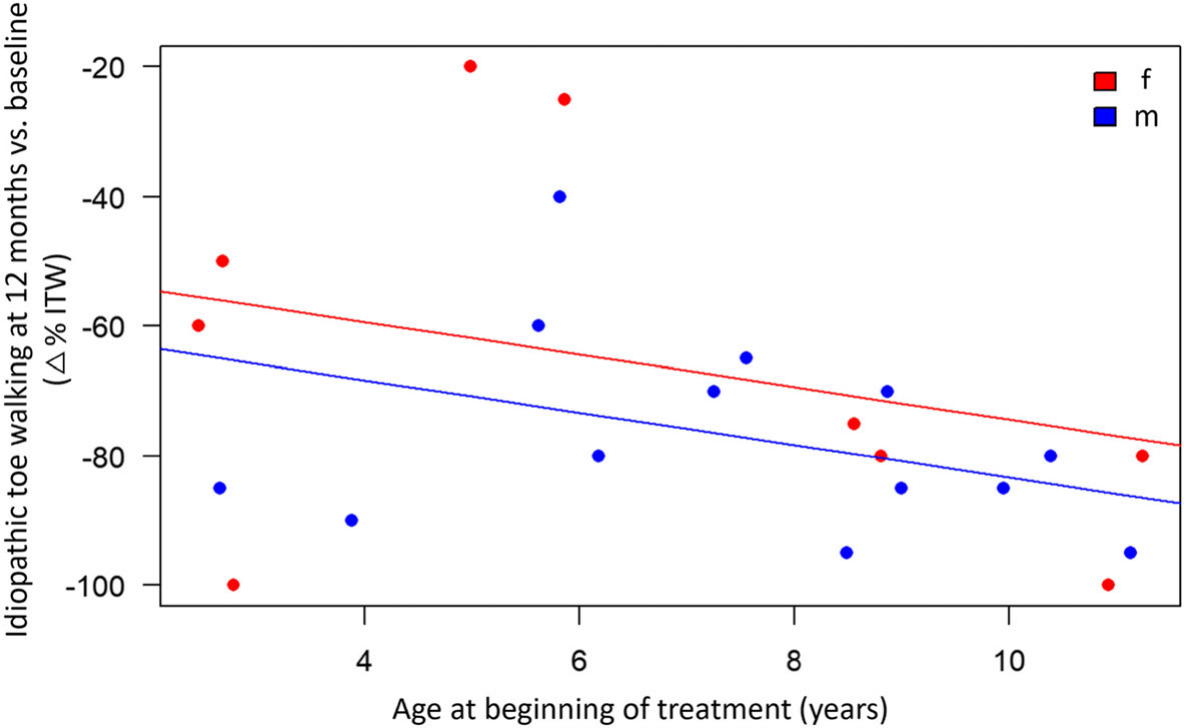
The multivariable linear regression shows a better improvement regarding percentage of ITW in the 12-months-follow-up for increased age at the beginning of treatment

After 12 months, a moderate positive correlation of toe walking improvement with age at the beginning of treatment (*r* = 0.38; *p* = 0.08) was seen. Looking at the multivariable linear regression, we recognized the adjusted effect that boys improved more (9.2%) than girls of the same age (*p* = 0.35). Within the sexes we found that older patients improved more (Fig. 6). Statistically, toe walking time at the 12 months follow-up was reduced by 2.5% with every year of age at the beginning of treatment (*p* = 0.13).

In seven patients the toe walking gait reocurred within 24 months. Four of them were males (24% of all boys) and three females (38% of all girls). Four 5-year-old patients (at the beginning of treatment) had to restart orthotic treatment within 24 months. The other three patients were between seven and 8 years old. Our results did not show any correlation between age and toe walking recurrence (*p* = 0.70).

The mean ankle dorsiflexion (DF) in an extended knee position was 3° +/− 7.5 (− 10° to 30°) at the beginning, improved to 16° +/− 4.8 (10° to 30°) at the end of treatment (or after treatment) and remained 14° +/− 7.5 (0° to 30°) after 12 months (Table 2).

**Table 2:** *%ITW* Percentage of idiopathic toe walking. ^a^one child (ID 12) developed recurrence shortly before the 12-months follow-up (%ITW_12_months). For statistical reasons, the last score of percentage of ITW and dorsiflexion before retreatment was applied

	**Patients’ data up to 12 months of follow-up**			
Patients (m/f)	22 (13/9)			
Mean age at beginning of treatment (yrs; range)	7,0 (2,5-11,2)			
Wearing time of orthoses (months)	22 (8-48)			
	Mean %ITW	Mean Dorsiflexion	Mean Grouping	Equal or less 50% tip toe walking (% of all patients)
Before treatment	83%	2.7°	4.7	*n* = 1 (5%)
0 months (end of treatment)	11%	16°	1.3	*n* = 22 (100%)
12 months	14%^a^	14°^a^	1.6	*n* = 21 (95%)

During orthotic treatment, six patients suffered mild pressure sores that did not result in an interruption of the treatment. None of the patients complained of pain while walking during the observation period. Other adverse effects were not reported.

## Discussion

Treatment of an idiopathic toe walking gait with lower leg orthoses resulted in gait normalisation in 73% of patients after 12 months (in the parents’ perception). Given the inaccurate nature of the data this result can only provide a tendency of the success of treatment. Compared to other studies also relying on parents’ perception, the results nevertheless seem promising.

In 2013 Engström et al. provided 51 idiopathic toe walkers with a lower leg cast for 4 weeks and additionally randomized 21 patients to botulinum toxin injections into the calf muscles beforehand. After 12 months, 12% (cast) and 30% (cast + botulinum toxin) of their patients showed a normalized gait pattern in parents’ perception [7]. Sätila et al. (2016) treated 30 children with stretching exercises for one-year, indoor shoes with solid heel caps and night-time splints made of a softcast, which had to be worn at least five nights a week. Sixteen of these patients received botulinum toxin injections into the calf muscles additionally. After 12 months, 28% (conservative group) and 56% (cons. + botulinum toxin) walked normally according to their parents, and 54 and 50% after 24 months, respectively [8].

ITW treatment with classical ankle foot orthoses was described by Herrin et al. in 2016 [10]. Ten subjects wore orthoses during daytime for 6 weeks. Gait analysis showed that the AFOs were 100% effective at preventing initial contact with the toe while being worn, but did not sustain this improvement in the follow-up condition with shoes only [10]. The parents did not notice a difference in time spent toe walking.

The lower leg orthoses investigated in our study seem to work for approximately three quarters of the patients within the first 12 months. One explanation might be that the biomechanical capacity of the lower leg orthosis is comparable to a walking cast, with the additional feature of a slight dorsiflexion in the ankle joint during every step. Similar to a cast, ankle mobility does improve with muscular stretching of both the soleus and the gastrocnemius muscles. The single articulated soleus muscle is stretched during the pre-swing phase of the walking cycle, when the foot is brought into dorsiflexion. Furthermore, knee extension will stretch the double articulated gastrocnemius muscle in mid stance. We know that cast treatment can lead to satisfying results in ITW, at least short-term. Thielemann et al. applied walking casts in maximum dorsiflexion for periods of 2 weeks until a minimum ankle ROM of 20° was obtained, to a group of ten patients aged 5 to 15 years [16]. After 6 months follow-up, all patients did maintain the capacity of dorsiflexion of more than 20°. Griffin et al. found a normalized gait pattern in six out of six patients immediately after serial casting in maximum dorsiflexion for 6 weeks [17]. Fox et al. followed up 44 patients with serial casting for 3-10 weeks, finding amelioration of ankle dorsiflexion of 5° after 14 months. Sixty-six percent of patients had stopped toe walking completely or enough to satisfy their parents [18].

Apart from muscular stretching, another reason might be that by preventing the toe walking gait for several weeks, the walking pattern and the dyspraxia does improve on a subconscious level [19–21].

During the night, while sleeping, wearing the orthosis might work by modulating sensory processing taking place via stimulation of proprioceptive and tactile receptors during subconscious movements. Deliberate night-time bracing was continued in our study group by 41% of the patients for an average of 19 weeks and seemed to have a positive influence on the gait pattern during daytime. A dysfunction of sensory processing has been associated with ITW in multiple studies in the past [22–26]. Williams et al. (2014) found a connection between partial dysfunction and hyposensitivity to tactile stimuli due to an immature or mild impaired cerebellum or motor cortex in ITW [27].

Not all patients in our study did completely benefit from the treatment with lower leg orthoses. After 12 months 18% (4/22) and after 24 months even 36% (8/22) of patients walked on the tip of their toes 50% or more of the day or had to be reequipped with an orthosis because of a recurrence. Contrary to previous results, older patients suffered less recurrences than younger ones in our small group of 22 patients The assumption exists that younger children may benefit more from serial casting [18].

Nevertheless, further interventions, for example procedures to the Achilles tendon, could be avoided in all patients during the observational period. It is questionable if more recurrences will occur in our cohort in the future. Genetic findings could play a role in this context.

### Limitations of the study

The main limitation of the study is the primary outcome measurement which is the perception of the parents and therefore obviously risky for bias. Smart wearables like socks with pressure sensors might provide the opportunity to measure daytime gait patterns objectively in the future. Retrospective observations could be confounded in general. The information value of our study is furthermore limited by the small number of cases and the absence of a control group. A randomized control trial including gait analysis should be planned in the future.

## Supplementary Information

**Additional file 1.**

## Data Availability

Details of the data are provided in the additional files section and can be obtained by the corresponding author via personal communication.
