# Supplementary material for: Orthotic treatment of idiopathic toe walking with a lower leg orthosis with circular subtalar blocking
Source: BMC Musculoskelet Disord. 2021 Jun 7;22:520. doi: 10.1186/s12891-021-04327-0 (PMC8183056; doi:10.1186/s12891-021-04327-0)
Supplement: Supplementary file 1 — Additional file 1. [file 12891_2021_4327_MOESM1_ESM.pdf]

Orthotic Treatment of Idiopathic Toe Walking with a Lower Leg Orthosis with Circular Subtalar Blocking

N. Berger, M. Bauer, A. Hapfelmeier, M. Salzmann, P.M. Prodinger

Patients' details

ID	SEX	Age at beginning of treatment (years)	Duration of treatment with orthoses (weeks)	Grouping at beginning of treatment	Dorsiflexion at beginning of treatment	Grouping immediately after end of treatment	Ankle dorsiflexion immediately after end of treatment	Grouping after 12 months of follow-up	Ankle dorsiflexion after 12 months of follow-up	Grouping after 24 months of follow-up	Ankle dorsiflexion after 24 months of follow-up	pain (other than pressure sores)	pressure sores by orthosis	Deliberate night-time bracing (weeks)
1	m	6,2	17,7	5	0	1	15	1	15	1	15	no	no	
2	f	2,7	12,0	3	5	1	20	1	20	1	20	no	no	
3	m	2,5	14,6	4	5	1	20	1	30	1	30	no	no	
5	m	3,9	13,0	5	0	1	15	1	15	1	15	no	no	
6	m	9,9	34,4	5	5	1	20	1	25	1	25	no	yes	18,4
8	m	7,3	47,9	4	-5	1	15	1	15	1	15	no	no	31,9
10	m	8,9	72,7	5	0	2	20	1	10	2	10	no	no	56,7
12	f	5,0	14,6	5	0	1	10	6	0 R	6	recurrence	no	no	
13	m	10,4	13,0	5	0	1	20	2	10	6	recurrence	no	no	
14	m	8,5	30,3	5	0	1	10	1	20	1	20	no	yes	14,3
15	f	10,9	8,0	5	0	1	10	1	10	1	20	no	yes	
16	m	2,6	8,6	5	5	1	20	1	20	1	20	no	no	
17	f	2,8	12,3	5	30	1	30	1	20	1	20	no	no	
18	m	7,6	14,6	4	0	1	15	1	5	6	recurrence	no	no	
21	m	5,6	28,3	5	5	1	20	3	10	6	recurrence	no	yes	12,3
22	m	5,8	15,1	5	15	2	15	3	5	6	recurrence	no	no	
26	m	11,1	27,0	5	0	1	20	1	20	1	20	no	yes	11,0
28	f	5,9	29,1	4	0	3	15	3	10	6	recurrence	no	no	13,1
30	f	8,8	13,0	5	0	1	10	1	15	1	20	no	no	
31	m	9,0	23,7	5	5	1	15	1	15	1	15	no	yes	7,7
32	f	8,6	20,6	5	-10	3	10	2	0	6	recurrence	no	no	4,6
33	f	11,2	16,9	4	0	1	15	1	20	1	20	no	no	

Supplementary data of patients' details: Duration of treatment with orthoses: summary of treatment time including deliberate night-time bracing. Grouping: group 1: 0% idiopathic toe walking (ITW), group 2: 25% ITW, group 3: 50% ITW, group 4: 75% ITW, group 5: 100% ITW, group 6: recurrence. Ankle dorsiflexion: maximum passive dorsiflexion capacity of the ankle joint in a straight leg position, measured as described in the main body of the text. Note: One child (ID 12) developed recurrence shortly before the 12-months follow-up. For statistical reasons, the last score of percentage of ITW and dorsiflexion before retreatment was noted. Some patients chose to continue night-time bracing after the standard curriculum had ended ("deliberate night-time bracing").
